# Stretch Formability of an AZ61 Alloy Plate Prepared by Multi-Pass Friction Stir Processing

**DOI:** 10.3390/ma14123168

**Published:** 2021-06-09

**Authors:** Xicai Luo, Haolin Liu, Limei Kang, Jielin Lin, Yifei Liu, Datong Zhang, Dongyang Li, Daolun Chen

**Affiliations:** 1 School of Electrical and Mechanical Engineering, Guangzhou Railway Polytechnic, Guangzhou 510430, China; meluoxicai@mail.scut.edu.cn (X.L.); liuhaolin@gtxy.edu.cn (H.L.); kanglimei@gtxy.edu.cn (L.K.); 202016243023@gtxy.edu.cn (J.L.); liuyifei@gtxy.edu.cn (Y.L.); 2 National Engineering Research Center of Near-Net Shape Forming for Metallic Materials, Guangdong Key Laboratory for Processing and Forming of Advanced Metallic Materials, School of Mechanical and Automotive Engineering, South China University of Technology, Guangzhou 510640, China; 3 Department of Chemical and Materials Engineering, University of Alberta, Edmonton, AB T6G 2H5, Canada; dongyang@ualberta.ca; 4 Department of Mechanical and Industrial Engineering, Ryerson University, 350 Victoria Street, Toronto, ON M5B 2K3, Canada

**Keywords:** microstructure evolution, stretch formability, Erichsen cupping test, multiple-pass friction stir processing

## Abstract

The stretch formability behavior of an AZ61 magnesium alloy plate produced by multi-pass friction stir processing (M-FSP) was investigated, with the applied load vs. displacement curves recorded during Erichsen cupping tests at different punching speeds at room temperature. The stretch formability of M-FSP AZ61 magnesium alloy was significantly enhanced, compared with that of its cast counterpart. The highest Erichsen index of 3.7 mm was obtained at a punching speed of 0.1 mm/min. The improved stretch formability was mainly attributed to the grain refinement stemming from the M-FSP and the presence of extension twinning to accommodate deformation during Erichsen cupping testing.

## 1. Introduction

Magnesium alloy, as a lightweight structural material, has the advantages of low density, high specific strength, good machinability and high recycling potential. It has thus received considerable attention for the potential applications in the aerospace, automotive and 3C industries. However, magnesium alloys with a typical hexagonal close-packed (HCP) crystal structure have a limited number of slip systems, which dramatically restrains their applications due to low formability and ductility at room temperature. Therefore, the majority of the magnesium alloy parts have been made by casting instead of wrought technologies [1,2]. However, casting defects are easily present in the cast magnesium alloys, resulting in low mechanical properties and thus being difficult to meet the requirements of structural applications. Grain refinement has been proven to be an effective method to improve the mechanical properties of magnesium alloys prepared by severe plastic deformation (SPD) techniques, such as equal-channel angular pressing (EACP), and high-pressure torsion (HPT). However, it is difficult for the EACP and HPT techniques to be widely used in the industrial applications due to the complex processes, high energy consumption, high cost, and limited sample size [3]. It is still a great challenge to develop a technique for manufacturing effectively large-size magnesium alloy plates with fine-grained structure. This is indeed one of the key barriers in promoting their applications in industry.

It is reported that rolling technology is a common method for preparing large-size magnesium alloy plates, while it creates some problems, i.e., lower efficiency, high cost, strong basal texture and poor formability, compared with rolled aluminum alloys [4]. Chino et al. [5] evaluated stretch formability of rolled AZ31 alloy sheets through Erichsen cupping tests at room temperature and elevated temperatures and observed that the reduction of strain and plastic anisotropy are associated with texture intensity and texture distribution, while fine grain size becomes an important factor for the stretch formability of magnesium alloy deformed at elevated temperatures. Huang et al. [6] investigated AZ61 magnesium alloy subjected to rolling in different conditions and reported that the improvement of stretch formability can be achieved through homogenizing microstructures with a relatively weaker texture due to rotating the inclination angle of the basal pole toward the rolling direction and weakening the basal texture during deformation.

Friction stir processing (FSP), as an alternative SPD technique, has been widely reported to be capable of achieving significant grain refinement and improved mechanical properties of magnesium alloys [7,8,9,10,11]. After FSP, the recrystallization texture becomes weaker due to the occurrence of recrystallization and materials flow around the rotating pin. However, one pass FSP just modifies a limited area dependent on the dimension of the pin, which restricts its application. Multi-pass friction stir processing (M-FSP) with a certain overlapping ratio has been used to successfully produce large-size lightweight alloys such as magnesium and aluminum alloys [12,13,14]. A lot of studies have been conducted to evaluate the effect of overlapping ratio, overlapping direction and processing parameters on the microstructural evolution, mechanical properties and superplasticity [15,16,17,18,19,20,21]. However, there is a lack of the stretch formability of large magnesium alloy plates prepared by the M-FSP. It is unclear if such manufactured plates are suitable for deep drawing and what fracture characteristics of M-FSP magnesium alloy plate would be in the Erichsen cupping test. The aim of this study is, therefore, to identify the effect of punching speed on the stretch formability and fracture behavior of M-FSP AZ61 magnesium alloy.

## 2. Materials and Methods

**Figure 1:**
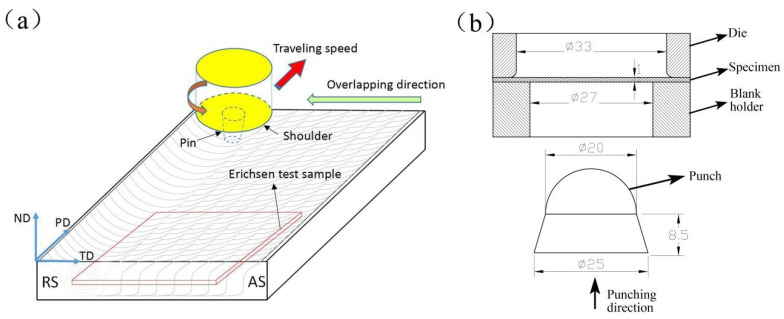
(**a**) Schematic illustration of multi-pass friction stir processing (M-FSP) plate showing the machining position of Erichsen cupping test samples; (**b**) experimental set-up of the stamping die.

AZ61 cast magnesium alloy with a composition of 6.8 wt.% Al and 0.79 wt.% Zn was selected in the present work. A plate with a length of 180 mm, width of 70 mm and thickness of 6 mm was machined from the cast alloy for FSP. Prior to FSP, the plate was ground with sandpapers up to a grit of #2000, and then cleaned with ethanol. FSP was carried out via a welding machine (FSW-3LM-003, FSW Technology Co. Ltd, Beijing, China) at a rotational rate of 1000 rpm and a welding speed of 60 mm/min. The subsequent pass of FSP was moved toward the retreating side with an overlapping ratio of 50%. In this manner, the plate with a total of 26 overlapping passes was prepared, as shown in Figure 1a. The stirring tool, with a shoulder of 18 mm in diameter and a threaded conical pin of 7 mm in root diameter and 5 mm in length, was constantly tilted toward the normal direction (ND) with an angle of 2.5°.

**Figure 2:**
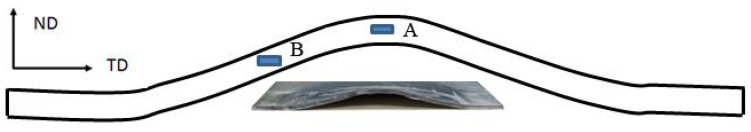
Locations of damped samples detected by electron backscatter diffraction (EBSD).

Macrostructures and microstructures of the M-FSP sample were examined via a scanning electron microscope (SEM, JSM-6380LV, JEOL, Japan) along with energy-dispersive spectroscopy (EDS, Inca300, Oxford, UK). The examined samples were prepared using standard metallographic techniques and etched with a picric acid solution consisting of 80 mL ethanol, 10 mL distilled water, 10 mL acetic acid and 5 g picric acid. The locations of tested samples on the transverse direction (TD) and ND cross-section detected by electron backscatter diffraction (EBSD) were shown in Figure 2. The EBSD samples were prepared through grinding, mechanical polishing and electro-polishing with a solution (60 mL ethanol, 15 mL acetic acid, 5 mL nitric acid and 20 mL stilled water) at 5 V for 6 s at room temperature. EBSD information was collected via an Oxford instrument EBSD detector (HKL-Nordlys, Oxford, UK) and the experimental data were analyzed through the HKL-Channel 5 (Version 5.12.62.0) software.

The specimens for Erichsen cupping tests were machined from the stirring zone of the M-FSP plate with a size of 40 × 40 × 1 (length × width × thickness, mm), as shown in Figure 1a. Before the test, the specimens were ground with sandpapers up to a grit of #2000. The Erichsen cupping tests were carried out in a machine (WBT-60B, AJ, Shanghai, China) with a lubricant of Vaseline to reduce the friction. The punching speed was set to be 0.1 mm/min, 1 mm/min, and 10 mm/min, respectively. The equipment for Erichsen cupping tests consisted of a punch of 20 mm in diameter (hemispherical), a pressure die of 27 mm in diameter and a blank holder of 33 mm in diameter, as shown in Figure 1b. The deformed macrostructures and microstructures of failed samples were also examined via SEM and EBSD.

## 3. Results

### 3.1. Stretch Formability of Multi-Pass Friction Stir Processing (M-FSP) AZ61 Plate

**Figure 3:**
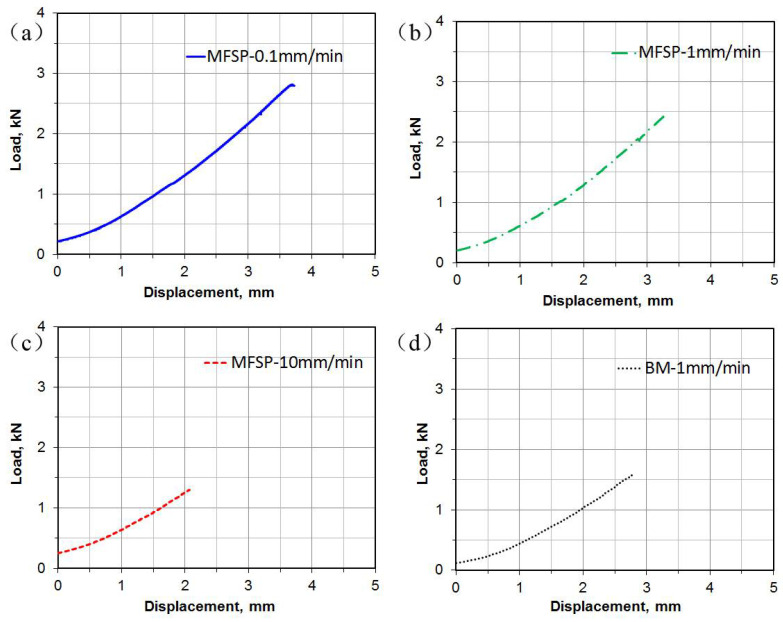
Typical load-displacement curves during Erichsen cupping tests at different punching speeds of (**a**) 0.1 mm/min; (**b**) 1 mm/min; (**c**) 10 mm/min for the M-FSP samples; and (**d**) at 1 mm/min for the base material (BM).

Figure 3 shows the load-displacement curves obtained from the Erichsen cupping tests at different punching speeds at room temperature. It is seen that the M-FSP sample exhibited an Erichsen index (IE) of 3.3 mm at a punching speed of 1 mm/min (Figure 3b), which was higher than that of base material (BM) (Figure 3d). This indicates that the formability of AZ61 alloy plate was improved by M-FSP. Also, the formability of M-FSP plate increased with decreasing punching speed. The highest IE value of 3.7 mm for M-FSP AZ61 plate was achieved in the Erichsen cupping test at a punching speed of 0.1 mm/min (Figure 3a), despite a lower IE value of 2.1 mm at a high punching speed of 10 mm/min as shown in Figure 3c. 

**Figure 4:**
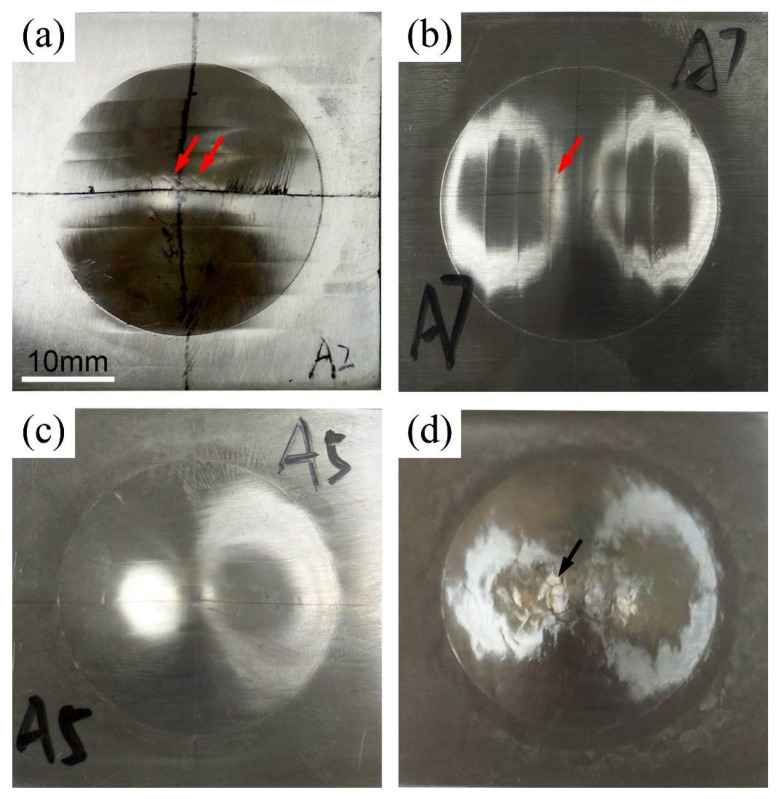
Macroscopic appearances of the M-FSP plates after Erichsen cupping tests at different punching speeds of (**a**) 0.1 mm/min; (**b**) 1 mm/min; (**c**) 10 mm/min; and (**d**) BM tested at 1 mm/min.

**Figure 5:**
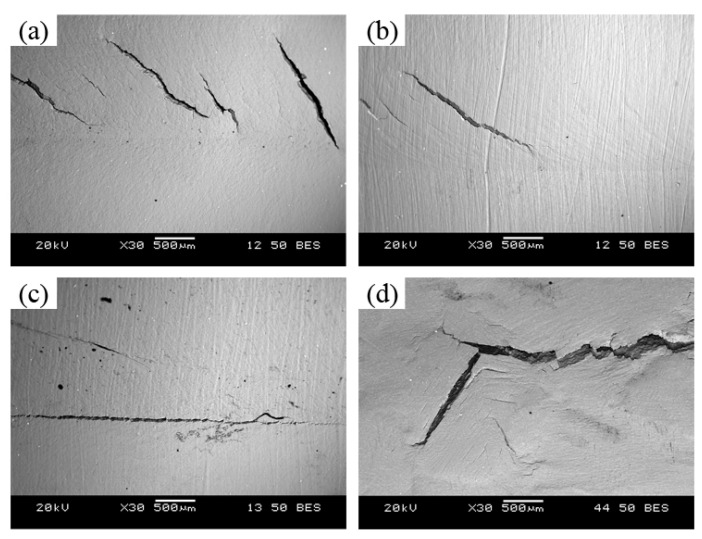
Crack propagation of M-FSP plates after Erichsen cupping tests at different punching speeds of (**a**) 0.1 mm/min; (**b**) 1 mm/min; (**c**) 10 mm/min; and (**d**) BM tested at 1 mm/min.

Figure 4 shows the macroscopic appearances of the BM and M-FSP plates after Erichsen cupping test at different punching speeds of 0.1 mm/min, 1 mm/min, and 10 mm/min, respectively. It is clearly seen that the appearances of the deformed samples presented overlapping profiles, resulting from the multiple passes processing. Moreover, some micro-cracks could be seen at the top of the cup, where a large amount of deformation was present. Close examinations at a higher magnification revealed the crack formation and propagation of M-FSP plates after Erichsen cupping tests at different punching speeds, as shown in Figure 5. It is noticed that the crack shapes were different, depending on the punching speed. More arc-shaped cracks appeared in the samples tested at low punching speeds of 0.1 mm/min and 1 mm/min, as shown in Figure 5a,b. Such cracks appeared to initiate and propagate along the onion-ring structure. As the punching speed reached 10 mm/min, the cracks initially generated and propagated along the interface of overlapping zones (Figure 5c). In the cast BM sample, the cracks appeared more openly and were in the form of intergranular cracking, as seen from Figure 5d.

### 3.2. Microstructural Evolution 

**Figure 6:**
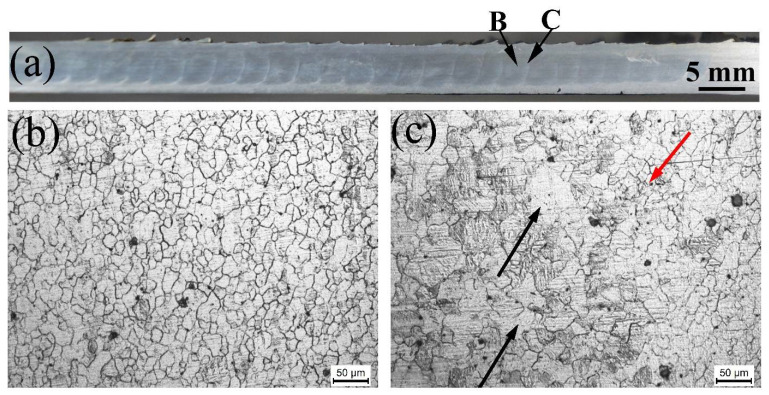
(**a**) A low-magnification macroscopic morphologies in the cross-section of M-FSP AZ61 alloy plate; optical micrographs at (**b**) Location B and (**c**) Location C in (**a**).

Figure 6a shows a typical cross-sectional macrograph of the M-FSP AZ61 plate, where different regions, i.e., stirring zones, transition zones, can be clearly seen. The processing profiles of each pass are present in the cross-section, as also reported in our previous paper [22]. The microstructure in location B marked in Figure 6a is shown in Figure 6b, where two passes of FSP were experienced, leading to more uniform and finer grain sizes with an average size of 10 μm. Location C in a transition zone between the seventh pass and eighth pass could be considered as the thermo-mechanically effected zone (TMAZ) of the subsequent FSP. Compared with location B, the non-uniform microstructures were present, consisting of coarse grains (marked by black arrows) and fine grains (marked by a red arrow), as shown in Figure 6c. This is attributed to the fact that the initial refined grains produced by the previous FSP pass were coarsened by the subsequent FSP pass due to additional heat input. 

**Figure 7:**
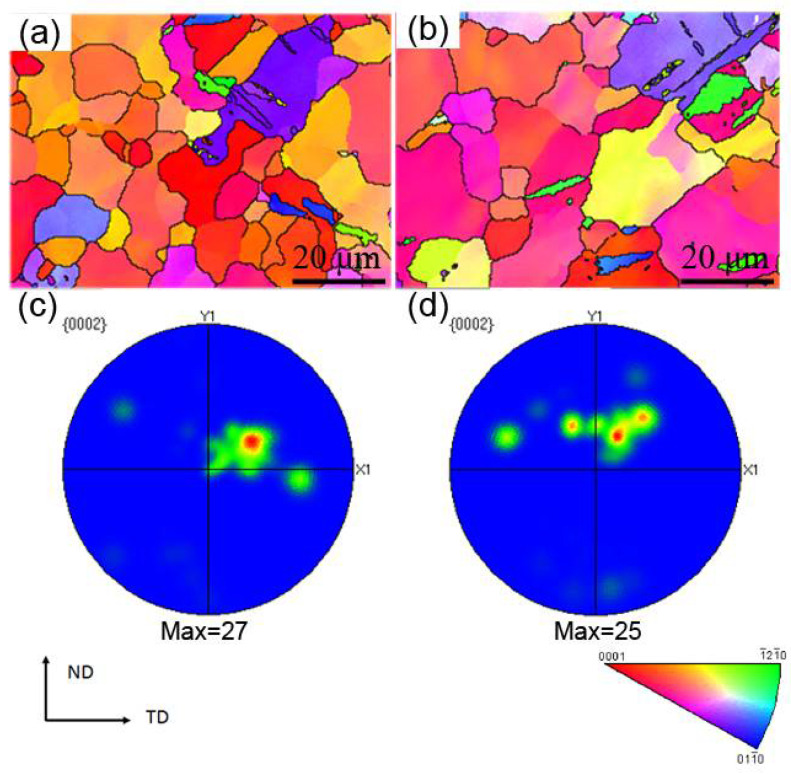
(**a**,**b**) Inverse pole figure (IPF) orientation maps, (**c**,**d**) {0002} pole figures, respectively, showing the corresponding microstructure and texture at locations A and B labelled in Figure 2a after Erichsen cupping test at a punching speed of 0.1 mm/min.

Figure 7 shows the EBSD orientation maps of a specimen after Erichsen cupping test at a punching speed of 0.1 mm/min. The failed specimen was cut in half with the EBSD observation positions of A and B marked in Figure 2. As seen from Figure 7a,b, the extension twinning occurred in some grains at both positions, which was verified by a misorientation of ~86° across the twin boundary. It is clear that these grains (mainly purple grains) had a favorable orientation with a high Schmid factor with respect to the principal stress direction. The presence of twinning can promote plastic deformation, thereby it is beneficial to improve the formability of the sheet [5,23,24,25]. Positions B and C are dominated by basal texture in accordance with the orientation color legend, and the pole figures (Figure 7c,d) with a fairly high texture intensity of ~25–27 MRD (multiples of a random density) in the deformed sample, which is not beneficial for further deformation. 

## 4. Discussion

### 4.1. Stretch Formability of the M-FSP AZ61 Alloy Plate

Compared with the cast BM plate, the formability of the AZ61 magnesium alloy plate prepared by M-FSP was improved due to grain refinement. The IE value of M-FSP AZ61 alloy plate tested at room temperature is comparable to that of a rolled AZ31 alloy (3~4 mm), while it is relatively lower than that of steel or aluminum alloy sheet [5]. The stretch formability of the AZ series magnesium alloy plates is summarized in Table 1. It is seen that the stretch formability of magnesium plates exhibits a large difference, depending on processing techniques; it is hard to obtain good formability for the AZ31 and AZ61 magnesium plates prepared by normal rolling technique [5,26]. An IE value of 7 mm was obtained by differential speed rolling at a high temperature of 520 °C [24]. Furthermore, the IE values are also dependent on the grain size produced by different processes, as shown in Table 1. The stretch formability of the present M-FSP AZ61 magnesium alloy plate with an IE value of 3.7 mm tested at a punching speed of 0.1 mm/min at room temperature was equivalent or slightly superior to that of AZ61 produced by differential speed rolling at 370 °C (with an IE value of 3.3 mm) [26]. It should be noted that the results are related to the grain size and non-uniform microstructure of the M-FSP magnesium alloy plate (Figure 6). Grain refinement is beneficial for the formability of M-FSP alloy plate compared with that of cast plate, which is in good agreement with the results reported by Sato et al. [27]. However, the effect of grain size on the formability is complex. Kang et al. [28] and Chino et al. [29] considered that coarse grains in the magnesium plate could also have a possibility to improve its formability because twins are easily generated during the deformation of coarse grains, which facilitate the lattice rotation and lead to random texture distribution. The stretch formability would decrease as the grain sizes in the magnesium plate exceed a certain extent, such as more than 16 μm [30]. 

The non-uniform microstructures generated by overlapping FSP passes could be harmful to the formability, because the crack could easily initiate from the transitional zone and propagate along the non-uniform microstructural band due to high local residual stresses reported in our previous paper [31]. As shown in Figure 4a,b, the arc-shaped cracks generally initialed and propagated along the microstructural band when the M-FSP plates were subjected to the Erichsen cupping test at a low punching speed (≤1 mm/min). Cao et al. [32] observed the fracture morphology of a failed tensile specimen of FSP magnesium alloy, and observed that the cracks propagated along the “onion-ring structure”. With increasing punching speed (10 mm/min), a straight crack appeared along the interface of TMAZ/AZ (Figure 5c), indicating that the crack initiated and propagated from interface even with a non-uniform microstructure. 

**Figure 8:**
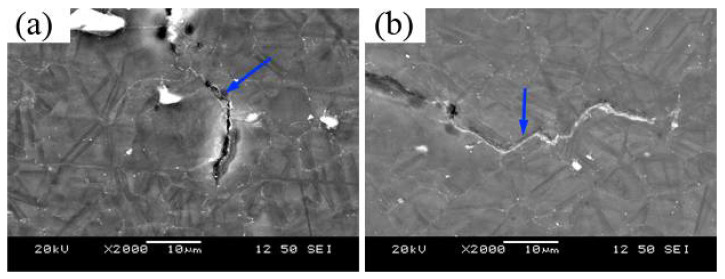
Crack propagation paths of M-FSP AZ61 plates during Erichsen cupping test at a punching speed of 0.1 mm/min: (**a**) along grain boundaries and (**b**) along twin boundaries.

In order to further clarify the fracture behavior of the stretch deformation, the crack propagation path was investigated for the M-FSP plate subjected Erichsen cupping test at a punching speed of 0.1 mm/min, as shown in Figure 8. It can be seen that some cracks initiated and propagated along the grain boundaries of coarse grains, as shown by the arrow in Figure 8a. Park et al. [30] observed the crack morphology of a ZX60 alloy after stamping through EBSD and SEM, and their results showed that the cracks are easy to initiate from the grain boundary and then propagate along the grain boundary. Figure 8b shows that a crack path along the twin boundary, which tends to generate in the coarse grains. Cracks initiated from the twins as the stress reached a certain level, and then propagated along the twin boundary and then grain boundary, which is in a good agreement with the results reported by Somekawa et al. [33].

### 4.2. Effect of Extension Twinning on the Stretch Formability of M-FSP Plate

The formability of a magnesium plate is closely related to texture in addition to grain size. As shown in Table 1, it can be seen that the plate with a low texture intensity exhibited a better formability. Huang et al. [24] reported that the AZ61 sheet rolled at a high temperature of 520 °C exhibited a superior stretch formability with an IE value of 7 mm at room temperature, due to the weakened basal texture regardless of coarser grains. The reason is that the randomly distributed or weakened texture is conducive to basal slip, and even facilitates the non-basal slip system to start in the process of magnesium alloy deformation [23,34]. In addition, the texture intensity and the inclination angle of the basal polar axis affect not only the activation of basal slip, but also the anisotropy of the plate [35]. Huang et al. [26] studied the effect of differential speed rolling parameters on an AZ61 magnesium alloy sheet, and revealed that the polar axis of the grains tends to orient in the rolling direction with the change of processing parameters, which is more conducive to stretch formability.

The texture intensity of the deformed specimen is high (Figure 7c,d), which is not beneficial for the formability. During deformation, the movement of dislocations continues to pile up at the grain boundaries leading to a stress concentration, thereby other deformation mechanisms need to coordinate for further deformation. Extension twinning was present in some grains of the deformed specimen, as shown in Figure 7. The extension twinning of the grains with a high Schmid factor with respect to the principal stress direction was easily activated at room temperature. When the resolved shear stress reached its critical value, twinning occurred mainly in the coarser grains, which resulted in an equivalent rotation of the c-axis of the crystal lattice (~86.3°) and thus changed the orientation in part of a grain [36,37,38]. The presence of twinning can accommodate further plastic deformation during forming, and thereby it is beneficial to improve the formability of magnesium alloy sheets [6,23,24,25].

## 5. Conclusions

The main conclusions of this study can be summarized as follows: multi-pass friction stir processing was applied on AZ61 cast alloys to produce a large-scale magnesium plate with non-uniform microstructures. The stretch formability of M-FSP AZ61 magnesium alloy was significantly improved compared with that of the cast base material, and the highest IE value of 3.7 mm was obtained at a punching speed of 0.1 mm/min at room temperature. Non-uniform microstructures of the M-FSP AZ61 plate affected the fracture behavior, with the cracks mainly initiated from the microstructural band at the overlapping interface. The presence of extension twinning is beneficial to the formability of an M-FSP AZ61 alloy plate. 

## Figures and Tables

**Table 1:** Erichsen index (IE) values of AZ series magnesium alloy plates prepared by different processes.

Materials	Processing	IE (mm)	Grain Size (μm)	Texture Intensity (MRD)	References
AZ31	Normal rolling	2.9	6.3	23.6	[5]
AZ31	Normal rolling	4.1	10.9	10.9	[5]
AZ31	Normal rolling	3.1	14.2	26.5	[5]
AZ31	Rolling at 550 °C + DSR at 225 °C	9.7	15	2.7	[25]
AZ61	DSR* at 520 °C	7.0	7.4~11.5	3.7	[24]
AZ61	DSR at 370 °C	3.3	5.2~11.5	7.0·	[26]

DSR*: Differential speed rolling.

## Data Availability

The raw/processed data required to reproduce these findings cannot be shared at this time as the data also form part of an ongoing study.
